# SiRNA-Mediated *RRM2* Gene Silencing Combined with Cisplatin in the Treatment of Epithelial Ovarian Cancer *In Vivo*: An Experimental Study of Nude Mice

**DOI:** 10.7150/ijms.33979

**Published:** 2019-10-21

**Authors:** Ting Xue, Liming Wang, Yong Li, Hao Song, Huijun Chu, Hongjuan Yang, Ailian Guo, Jinwen Jiao

**Affiliations:** 1 Qingdao University, Qingdao, China.; 2 Department of Obstetrics and Gynecology, The Affiliated Hospital of Qingdao University, Qingdao, China.; 3 Department of Oncology, The Affiliated Hospital of Qingdao University, Qingdao, China.

**Keywords:** RRM2, siRNA/RNAi, cisplatin, ovarian cancer, nude mice

## Abstract

**Introduction**: We aimed to explore small interfering (si)RNA silencing of ribonucleotide reductase M2 (*RRM2*) gene combined with cisplatin for the treatment of human ovarian cancer in nude mice models of subcutaneous transplantation of tumor cells.

**Methods**: After conventional cultivation of human ovarian cancer cell line SKOV3 *in vitro*, SKOV3 cells were injected into the right back of nude mice by subcutaneous injection to establish the subcutaneous tumor models. Twenty-four tumor-burdened rats were randomly divided into four groups (n=6): siRNA group, siRNA in combination with cisplatin group, cisplatin group, and control group. Intraperitoneal injection of cisplatin and subcutaneous injection of siRNA were performed weekly. Tumor volume was measured, and tumor growth inhibition rate was calculated. *RRM2* expression at the mRNA and protein levels was detected by reverse transcription-polymerase chain reaction and immunohistochemistry.

**Results**: In the siRNA group, the tumor volume and tumor growth inhibition rate were 249.60±20.46 mm³ and 36.39%, respectively. The tumor growth inhibition rate and tumor volume were significantly different between the siRNA and control groups (p<0.05). In the cisplatin group, the tumor volume and tumor growth inhibition rate were 249.86±12.46 mm³ and 41.10%, respectively. The tumor growth inhibition rate and tumor volume were significantly different between the cisplatin and control groups (p<0.05). In the siRNA + cisplatin group, the tumor volume reduced to 180.84±16.25 mm³ and the tumor growth inhibition rate was increased to 64.33%, which were significantly different compared with the control group (p<0.01). Significant downregulation of *RRM2* mRNA and protein expression in the tumor tissues was detected by reverse transcription polymerase chain reaction and immunohistochemistry assay (p<0.05).

**Discussion**: siRNA alone or combined with cisplatin can effectively inhibit the growth of human ovarian cancer in nude mice models of subcutaneous transplantation of tumor cells. *RRM2* gene silencing may be a potential treatment regimen for ovarian cancer in future.

## Introduction

Ovarian cancer is one of the three most common malignancies of the female reproductive system, with the highest mortality rate among all gynecological tumors^1^. Surgery, combined with platinum- or paclitaxel-based chemotherapy, is the main treatment for patients with ovarian carcinoma. However, patients with ovarian carcinoma easily develop drug resistance^2^, such as cisplatin resistance. These drug-resistant patients generally have few treatment options. Therefore, there is an urgent need for the identification of novel therapeutic strategies targeting drug-resistant mechanisms to enhance cisplatin's killing effect on tumor cells and to increase sensitivity to chemotherapy.

Gene therapy refers to the introduction of exogenous genes into target cells to correct or compensate for diseases caused by genetic defects or abnormal gene expression. Innovations of gene therapy technology and clinical trials have increased in recent years, and a number of gene therapy projects have been approved and listed in the United States, China, and other countries to treat diabetes, cardio-cerebrovascular disease, rheumatism, and various types of cancer^3^-^9^. Ribonucleotide reductase (*RR*) is a potential therapeutic target for cancer because its role in catalytic reduction is necessary for DNA replication and repair^10^. It is the rate-limiting enzyme in the conversion of ribonucleotide 5′-diphosphates into 2′-deoxyribonucleotides. Human *RR* consists of two parts: *RRM1* and *RRM2*. Unlike *RRM1*, *RRM2* is only expressed during the late G1/ early S phase of the cell cycle, when DNA replication occurs^11^. Over expression of *RRM2* plays a positive role in tumor growth. Elevated RR activity and over expression of *RRM2* significantly increase the drug-resistant properties and the angiogenesis of human cancer cells^12^. *RRM2* was identified as a diagnostic marker of several cancers, suggesting that *RRM2* is a potential therapeutic target. Therefore, an anti-tumor strategy that interferes with the activity of *RRM2* has the potential to inhibit the growth of ovarian cancer. In our previous study^13^, our results suggested that small interfering RNA(siRNA)-mediated *RRM2* knockdown significantly reversed SKOV3/DDP cell resistance to cisplatin. Choosing an efficient gene delivery system has been a major challenge for gene therapy. We used Lipofectamine 2000 to effectively transfer siRNA into SKOV3/DDP cells. Previously, we have demonstrated the synergistic inhibitory effect of RNA interference technology combined with gemcitabine and cisplatin in SKOV3/DDP cells; however, no study has explored whether *RRM2* gene therapy can also reverse ovarian cancer resistance to cisplatin *in vivo*. Here, we used the human ovarian carcinoma SKOV3 cell line to construct a nude mouse subcutaneous transplantation model to investigate whether *RRM2* gene therapy was a novel therapeutic option for the treatment of epithelial ovarian cancer.

## Methods

### Cell culture

SKOV3 cell lines were purchased from the Cell Resource Center of the Shanghai Institute of Life Sciences and preserved by our laboratory. They were cultured in DMEM-F12 medium supplemented with 5% FBS, 100 μg/mL streptomycin, 100 U/mL penicillin, and 2 mM L-glutamine at 37°C in an incubator containing 5% CO_2_.

### siRNA duplexes

siRNA targeting *RRM2* -(sense: 5′-GGAGCGAUUUAGCCAAGAATT-3′; antisense: 5′-UUCUUGGCUAAAUCGCUCCTT-3′) was purchased from GenePharma (Shanghai, China) and a negative control siRNA was a gift from them.

### Lipofectamine transfection

Cells were seeded in 24-cell plates 24 hours before transfection in medium containing 10% FBS, so that they reached about 50% confluency. siRNA was complexed with Lipofectamine 2000 (Invitrogen, Carlsbad, CA, USA) according to the manufacturer's instructions and was applied to each control plate. These cells were divided into four groups: the blank group, the liposome group, the non-targeting siRNA group and the targeting siRNA group. Transfection media was removed and replaced with new media after 4 hours. Cells were collected after 72 hours and RNA was extracted for analysis.

### Animal procedures and treatment

**Figure 1:**
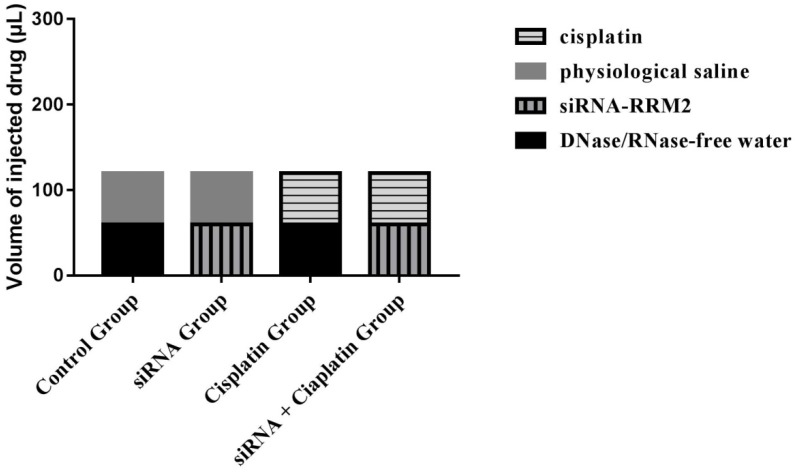
The specific administration methods of the four treatment groups.

All animal procedures were conducted in accordance with institutional and national guidelines. All experimental protocols were approved by the Animal Care and Welfare Committee of the Affiliated Hospital of Qingdao University. (License NO. AHQU20170914A) Female BALB/c nude mice (aged 4 weeks) were purchased from SHANGHAI SLAC and housed under specific pathogen-free conditions at the laboratory animal room for a week before the experiment. All of the mice were inoculated with a subcutaneous injection of 2 × 10^7^ cells plus PBS in the right dorsum (injection volume = 200 μL). The sizes of tumors were measured from the first day until the day of death after cell injection using calipers with the formula: V (volume) =1/2 × a × b^2^, where “a” represents the greatest length and “b” represents the perpendicular width^14^. Furthermore, tumor growth inhibition rate was calculated as: Tumor growth inhibition rate (%) = (tumor volume in control group - tumor volume in treatment group) / tumor volume in control group × 100%. When palpable tumors had developed at the sites of injection (>50 mm³), tumor-bearing animals were randomly allocated to four groups (n=6) and were treated with DNase/RNase-free water, cisplatin (3 mg/kg), physiological saline, and siRNA-*RRM2* (500 pmol) via intraperitoneal and subcutaneous injection after tumor inoculation, the specific administration methods of the four treatment groups were shown (Figure 1). Drug treatment was performed weekly for 4 weeks. All mice were sacrificed by cervical vertebra dislocation at 24 days after first dosage. Tumors were harvested and immobilized with 4% neutral paraformaldehyde and frozen with liquid nitrogen immediately. Tumor volume, number of nodules, and nude mouse weight were recorded every three days.

### Reverse transcription-polymerase chain reaction

Total RNA was extracted from the transfected cells and tumor tissues using RNAiso PLUS. The sample was reverse transcribed using a TaKaRa RNA PCR Kit (AMV) Version.3.0 (TaKaRa, Beijing, China). The *GAPDH* gene was used as an endogenous control. Primers were synthesized by Sangon Biotech (Shanghai, China) as follows: RRM2-Forward: 5′-GCGATTTAGCCAAGAAGTTCAGAT-3′, RRM2-Reverse: 5′-CCCAGTCTGCCTTCTTCTTGA-3′; GAPDH-Forward:5′-TCACTGCCACCCAGAAGACT-3′, GAPDH-Reverse: 5′-TTCTAGACGGCAGGTCAGGT-3′. The reverse transcription-polymerase chain reaction process contained a step at 94°C for 180 s, followed by 30 s at 94°C, 30 s at 57°C, and 45 s at 72°C for 32 cycles, followed by analysis.

### Immunohistochemistry

To detect intracellular localization and expression levels of *RRM2*, we used rabbit anti-human *RRM2* antibody (Abcam, ab209995, Tris-EDTA buffer) as the primary antibody, then combined it with a secondary antibody. Cell nuclei were counterstained using 4,6-diamidino-2-phenylindole (DAPI, Invitrogen). All tissue slides were evaluated and scored by a qualified pathologist. The expression of *RRM2* was determined by cytoplasmic staining intensity and positive cell rate. According to the staining intensity, the results were as follows: no staining (0), weak staining (1), medium staining (2), and strong staining (3). The positive cell rate was graded as < 5% (0), 6%~25% (1), 26%-50% (2), and > 50% (3). The final score is the sum of the above two scores.

### Statistical analysis

All data were presented as mean values ± standard deviation. The statistical significance was evaluated by one-way analysis of variance when all groups were compared, and Tukey's HSD for post-hoc analysis between two groups. In all tests, differences were considered to be statistically significant at p<0.05.

## Results

### RNA interference experiments *in vitro*

**Figure 2:**
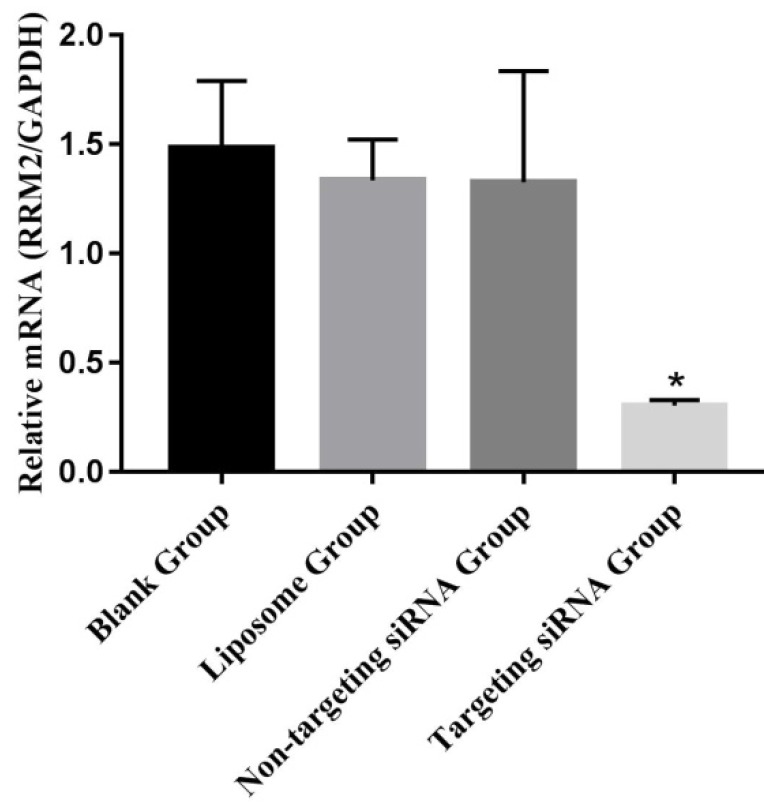
Expression of *RRM2* mRNA in SKOV3 cells. Relative mRNA level of *RRM2* in SKOV3 cells were analyzed by reverse transcription-polymerase chain reaction with *GAPDH* as a control, ^*^P<0.05 as compared with the blank group.

The expression levels of *RRM2* were examined with reverse transcription polymerase chain reaction in SKOV3 cells (Figure 2). *RRM2* mRNA was higher from the liposome group, the non-targeting siRNA group and the blank group than in the targeting siRNA groups (p<0.05). There was no significant difference between the blank and non-targeting siRNA groups (p>0.05).

### Tumor volume and tumor growth inhibition of subcutaneous transplanted tumors

**Figure 3:**
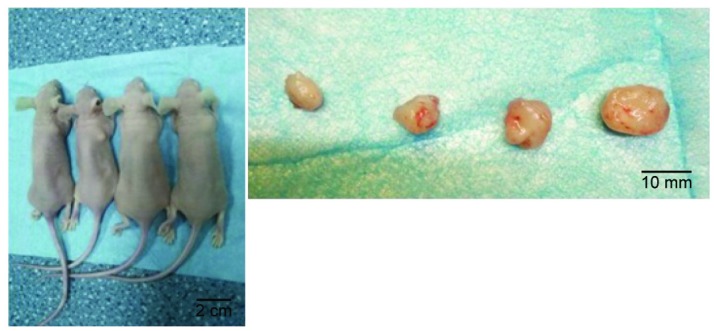
Model of subcutaneous transplanted tumors in nude mice. Subcutaneous transplanted tumors (left) in a whole animal and excised tumor tissues (right). From left to right: siRNA+ cisplatin group, cisplatin group, siRNA group, and control group.

**Figure 4:**
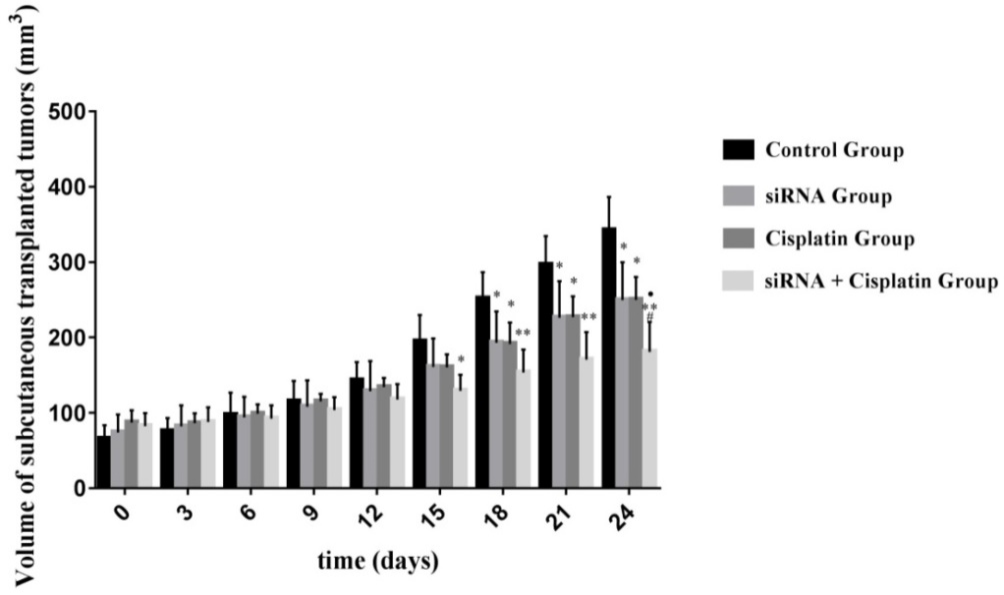
Volume of subcutaneous transplanted tumors on Nude Mouse at different time points. The sizes of tumors were measured from the first day until the day of death after cell injection using calipers with the formula. ^*^P<0.05, ^**^P<0.001 as compared with control group. ^Ÿ^P<0.05 as compared with siRNA group. ^#^P<0.05 as compared with cisplatin group.

Although treatment with siRNA or siRNA + cisplatin significantly suppressed tumor growth compared with that in the control group, the optimal therapeutic effect on tumor growth was achieved by siRNA + cisplatin treatment (Figure 3). The suppression of tumor growth in siRNA + cisplatin mice continued until the day of sacrifice, reaching a mean volume of 180.84 mm³, while tumors of mice treated with control, cisplatin, or siRNA grew persistently with mean tumor volumes of 342.13 mm³, 249.86 mm³, and 249.60 mm³, respectively (p<0.05). Furthermore, the combined treatment of siRNA and cisplatin caused marked tumor growth suppression compared with siRNA alone (p<0.05), but there was no significant difference between the siRNA and cisplatin groups (p>0.05; Figure 4).

### Pathological sections of tumor tissue

**Figure 5:**
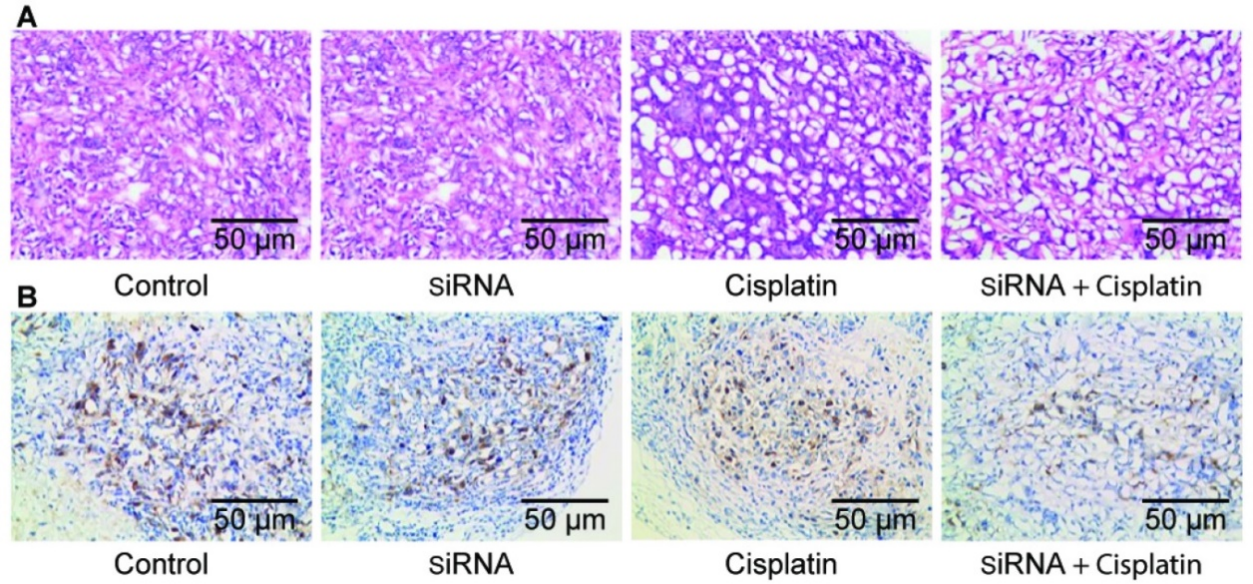
Pathological section of tumor tissue Histological examination with hematoxylin and eosin staining (A) and immunohistochemical staining (B) of tumor tissue.

After the nude mice were sacrificed, fresh tumors were excised. The tissue was fixed in formaldehyde solution and routinely made into paraffin sections. Histological examination with hematoxylin and eosin staining of tumor tissues showed necrotic cells along with tissue disorganization, with large tumor cells, large and hyperchromatic nuclei, prominent nucleoli, and obvious mitotic images in all treatment groups, especially in the siRNA + cisplatin group (Figure 5A). Immunohistochemistry showed expression of *RRM2* in the transplanted tumor tissues of each group, and cell staining was observed in each treatment group. The positive cells exhibited yellowish brown granules in the cytoplasm. The siRNA, cisplatin, and siRNA + cisplatin groups showed incomplete cytoplasmic expression, which was significantly higher in the control group (Figure 5B).

### Effects of *RRM2* with cisplatin therapy on expression of *RRM2* mRNA and protein in subcutaneous transplanted tumors

**Figure 6:**
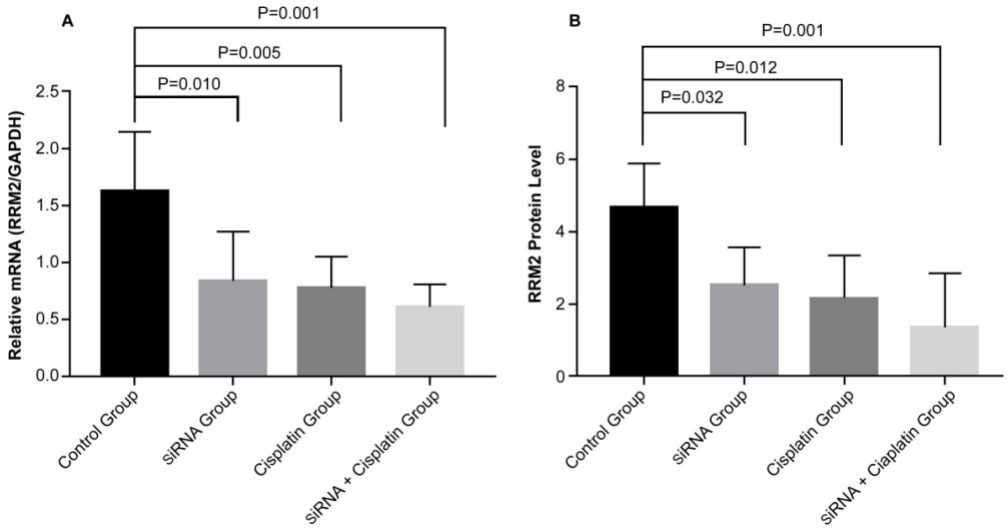
Expression of *RRM2* mRNA and related proteins in subcutaneous transplanted tumors. Relative mRNA level of *RRM2* in tumor tissues were analyzed by reverse transcription-polymerase chain reaction (A) with *GAPDH* as a control. The protein expression of *RRM2* in tumor tissue was assessed by immunohistochemical staining (B). All data were representative of three independent experiments.

To determine the potential mechanism of cell growth inhibition in subcutaneous transplanted tumors, the expression of *RRM2* mRNA and protein was examined. The gene and protein expression levels of *RRM2* were examined with reverse transcription polymerase chain reaction (Figure 6A) and immunohistochemical staining (Figure 6B) in subcutaneous transplanted tumors after different treatments. *RRM2* mRNA and protein expression was lower in tumors from the siRNA and siRNA + cisplatin groups than in the tumors of mice in the control groups and was significantly lower in the tumors of mice treated with siRNA + cisplatin, than in those from any other group (p<0.05). Compared with the control group, the mRNA and protein expression of *RRM2* in cisplatin group were lower (p<0.05). There was no significant difference between the siRNA and cisplatin groups (p>0.05).

## Discussion

Ovarian cancer is a malignant tumor that seriously endangers women's health. It has the highest mortality rate among gynecological tumors. Extensive pelvic and abdominal implantation and metastasis can occur in the early stages. Gene therapy is a new technology developed in recent years. As one of them, RNA interference mainly uses double-stranded RNA to specifically mediate the degradation of its complementary homologous mRNA series, so it can specifically inhibit the expression of the target protein with strong inhibition and high specificity. The combination of gene and chemotherapeutic drugs presents a promising therapeutic strategy for effective cancer treatment^15^-^18^. *RRM2* is not only a potential molecular marker of many malignant tumors, but also can disrupt the growth and differentiation of normal cells, thus playing the role of oncogenes^7^, ^19^, ^20^. *RRM2* interacts with many oncogenes to determine the potential for cell transformation and tumorigenesis. Intracellular RRM2 expression and enzyme activity are positively correlated with tumor resistance, invasion, and migration^21^, ^22^. Overexpression of *RRM2* can promote the proliferation, invasion, and drug resistance of oncogenes and increase the metastasis of tumor cells. In contrast, downregulation or silencing of the expression of *RRM2* can lead to apoptosis of malignant tumor cells, thereby inhibiting cell proliferation, metastasis, and reversing cell resistance^8^. Therefore, the expression level and activity of *RRM2* are closely related to the proliferation of tumor cells and may play a decisive role in the mechanism of controlling the invasion and development of malignant tumors.

In this study, we provided a novel strategy for ovarian carcinoma. Here, we used ovarian cancer cell line SKOV3 cells to construct subcutaneous transplanted tumor model. siRNA is used to treat tumor as a monotherapy or in combination with cisplatin. The relative low dose of cisplatin used in the present study did not produce obvious toxic effects, while those of siRNA are not yet clear. However, these levels were able to inhibit tumor growth in subcutaneous transplanted tumor when combined with *RRM2* gene therapy. High expression of *RRM2* is common in cancer and is also associated with resistance to chemotherapy and radiotherapy^23^, ^24^. Thus, this phenomenon led us to hypothesize that the expression level of *RRM2* is involved in the acquisition and development of resistance to multiple drugs. In a previous study, the increased sensitivity of SKOV3/DDP cells to cisplatin drugs after transfection with *RRM2* siRNA further demonstrated that *RRM2* may be an important mediator of cisplatin- mediated resistance^13^. We also further investigated the role of *RRM2* in tumor growth inhibition in subcutaneous transplanted tumors. In our study, compared with other groups, the expression of *RRM2* mRNA and protein and subcutaneous transplanted tumors volume were the lowest after siRNA + cisplatin treatment of mouse tumors. Resistance to apoptosis is a major reason for the failure of treatment of malignancies. Indeed, decreased *RRM2* levels induced by *RRM2* gene therapy may activate apoptosis pathways and inhibit cisplatin-induced DNA damage repair^23^, thereby promoting the apoptosis process initiated by anti-cancer agents. *RRM2* plays a key role in the regulation of DNA synthesis and cell proliferation in the DNA replication stage, mainly in the late G1 or early S phase of the cell cycle^25^, ^26^. In our study, we speculated that the slow growth of tumor volume in the siRNA + cisplatin group was related to the role of *RRM2*. On the one hand, the expression of *RRM2* was reduced, which arrest cell cycle at G1/S period and eventually induced necrosis. On the other hand, cisplatin leads to DNA damage. Inhibition of *RRM2* may reduce the DNA repair ability through blocking DNA repairing or lead to apoptosis when DNA repair is failed. The resistance to cisplatin is associated with the cell's increased ability of DNA repair. The combined treatment of siRNA and cisplatin caused marked tumor growth suppression compared with that of cisplatin alone, which may be related to siRNA increasing the sensitivity of tumor cells to cisplatin chemotherapy. One of the major problems of malignant progression is induction of invasion and migration. In addition to participating in DNA synthesis, *RRM2* also has an impact on the potential biological behavior and metastasis of malignant tumors and the generation of tumor drug resistance. If *RRM2* gene therapy combined with chemotherapy is an effective method for increasing the sensitivity of resistant cancer cells to chemotherapeutic agents, it will efficiently reduce the recurrence of invasion and migration. Previous studies have demonstrated that the *RRM2* gene plays a role in regulating tumor cell proliferation in different cancers and that decreased expression of *RRM2* increases sensitivity to temozolomide, gemcitabine, and platinum-based antitumor drugs^11^, ^25^, ^27^. Furthermore, studies have shown that several signal transduction pathways (such as VEGF, MMP2, and MMP9) are also associated with invasion and migration. Most human tumors overexpress VEGF, which is known to be a highly regulated angiogenic factor in cancer development^28^-^30^. Overexpression of *RRM2* increased VEGF expression. Knockdown of *RRM2* by siRNA may potentially inhibit cancer angiogenesis. MMPs involved in the cleavage of cell surface receptors possess gelatinase activity to enhance cancer invasion and metastasis^31^-^33^.

In this study, there was no significant injury to the lungs, kidney, or other important organs after the intraplastic injection of siRNA-*RRM2* directly into a transplanted tumor, indicating that siRNA-*RRM2* does not cause serious adverse reactions. However, the invasion of tumor cells was not tested in this study. Cunjian et al^34^. Subcutaneously inoculated an ovarian cancer cell line, SKOV3/DDP, into the necks of nude mice, and, in certain mice, metastatic lesions were found in the abdominal organs, including the liver and mesentery. Invasion and metastasis of tumor cells are mainly determined by their biological characteristics, as well as by factors such as the local microenvironment and host immunity. Tumor-bearing mice are mutant mice with congenital thymus defects. T lymphocyte development is blocked, resulting in T lymphocyte immune deficiency. The spleen is a key immune organ of nude mice, and other organs, such as the liver, also have a large number of macrophages, but no obvious tumor cell infiltration was found in our study.

As a limitation of our study, demonstrating the efficacy of this treatment in one cell line does not fully demonstrate its efficacy in other ovarian cancer cell lines expressing *RRM2*. Although our previous studies have shown that *RRM2* gene therapy may reduce the proliferation of SKOV3 cells *in vivo* and lead to an increase in apoptosis, the role of *RRM2* gene therapy in different ovarian cancer types still requires further research. Furthermore, methods for ensuring high efficiency, stability, and safety of gene therapy; selecting the appropriate transporter; and applying this research to clinical practice remain to be determined.

In conclusion, our study suggests that in a mouse subcutaneous transplanted tumor model, expression of *RRM2* mRNA and protein and the volume of subcutaneous transplanted tumors were the lowest in tumors of mice treated with siRNA + cisplatin. These results enhance our current understanding of the role of *RRM2* in tumor growth and provide new avenues for the development of effective treatment and prevention of ovarian cancer.

## Abbreviations

RNA: ribonucleic acid
DNA: deoxyribonucleic acid
siRNA: small interfering RNA
RR: ribonucleotide reductase
RRM2: ribonucleotide reductase M2
mRNA: messenger RNA
SKOV3/DDP: cisplatin-resistant SKOV3 cell
RNAi: RNA interference
VEGF: vascular endothelial growth factor
MMP: matrix metalloproteinase
DMEM-F12: dulbecco modified eagle medium mix with Ham's F12 nutrient medium
FBS: fetal bovine serum
Tris-EDTA buffer: buffer containing trimethylolamine, ethylene diamine tetraacetic acid and Tween 20, PH9.0
PBS: phosphate-buffered saline
